# Association between right ventricle two- and three-dimensional echocardiography and exercise capacity in patients with reduced left ventricular ejection fraction

**DOI:** 10.1371/journal.pone.0199439

**Published:** 2018-06-21

**Authors:** Aleksandra Sljivic, Milena Pavlovic Kleut, Zoran Bukumiric, Vera Celic

**Affiliations:** 1 Department of Cardiology, University Clinical Hospital Center "Dr. Dragisa Misovic—Dedinje", Belgrade, Serbia; 2 Institute for Medical Statistics and Informatics, Faculty of Medicine, University of Belgrade, Belgrade, Serbia; 3 Faculty of Medicine, University of Belgrade, Belgrade, Serbia; Ospedale del Cuore G Pasquinucci Fondazione Toscana Gabriele Monasterio di Massa, ITALY

## Abstract

**Introduction:**

Echocardiography represents the most commonly performed noninvasive cardiac imaging test for patients with heart failure (HF). The aim of this study was to assess the relationship between exercise capacity parameters (peak oxygen consumption (VO_2_) and the minute ventilation-carbon dioxide production relationship (VE/VCO_2_)), two-dimensional speckle-tracking echocardiography (2D-STE) and three-dimensional echocardiography (3DE) imaging of right ventricular (RV) function in HF patients with reduced ejection fraction (EF).

**Material and methods:**

This cross-sectional study included 54 patients with diagnosed ischemic LV systolic dysfunction (HF with reduced EF <40%) divided in subgroups based on the proposed values of the analyzed cardiopulmonary exercise testing (CPET) variables: VO_2_ peak ≤ 15 ml/kg/min, VO_2_ peak > 15 ml/kg/min, VE/VCO_2_ slope < 36 and VE/VCO_2_ slope ≥ 36. All patients underwent a physical examination, laboratory testing, conventional echocardiography, 2D-STE, 3DE, and CPET.

**Results:**

RV fractional area change (FAC), 2D RV global longitudinal strain (GLS), 3D RV EF were significantly decreased, and RV basal diameter (BD), systolic pulmonary artery pressure (SPAP), tricuspid annular plane systolic excursion (TAPSE), ratio between tricuspid flow and tissue Doppler derived e’ of the lateral tricuspid annulus (TV E/e’) were significantly increased in the subgroups of subjects with a worse VO_2_ peak and VE/VCO_2_ slope values. There was a significant positive correlation between the peak VO_2_ values and TAPSE, 2D RV GLS, 3D RV SV, and 3D RV EF as well as a significantly inverse correlation with VE/VCO_2_ slope.

**Conclusions:**

The observed significant correlation between the examined parameters suggests that 2D RV GLS and 3D RV EF, SV are associated with exercise capacity in patients with reduced HF.

## Introduction

In the past, the physiological importance of the right ventricle (RV) was underestimated. It used to be regarded as a neglected or forgotten chamber of the heart which mainly functions as a conduit; whereas its contractile performance was thought to be hemodynamically unimportant and less relevant in cardiac diseases than its left counterpart [1–3]. However, recent findings have showed that the RV plays an important role in the morbidity and mortality of patients with congestive heart failure, arrhythmia, ischemic heart disease, sudden cardiac death, pulmonary hypertension, etc. [4–6].

The assessment of RV dimensions and its function may be achieved by different methods available, including right cardiac catheterization, X-ray computed tomography, cardiac magnetic resonance (CMR) imaging, and echocardiography [7]. Although generating an accurate assessment of the RV function is a difficult and challenging task in clinical practice due to the complex geometry and retrosternal position of the RV, but also due to its interdependency with the left ventricle, echocardiography still represents the mainstay of evaluation of the RV structure and function. In comparison to other methods, echocardiography is widely available, less expensive, noninvasive, and requires no radiation or contrast injection [7].

Exercise intolerance is implicated in the heart failure (HF) syndrome. Cardiopulmonary exercise testing (CPET) has an important role in the assessment of HF status [8, 9], especially because a wealth of previous investigations have consistently demonstrated its prognostic values in the HF population [10, 11]. According to recent guidelines [8, 9] and the results of the conducted meta-analyses [10, 11], peak oxygen consumption (VO_2_), the minute ventilation-carbon dioxide production (VE/VCO_2_) relationship, the oxygen uptake efficiency slope (OUES), rest and peak pulmonary end-tidal carbon dioxide pressure (PETCO2) and exercise oscillatory ventilation (EOV) have been the most frequently assessed variables obtained from CPET. Peak VO_2_ and VE/VCO_2_ slope were the first CPET variables used in clinical practice, and they are considered to be the diagnostic and prognostic gold standard in HF patients [10, 11].

Previous studies have investigated the association between the parameters of RV function assessed by radionuclide ventriculography (RNV) techniques and exercise capacity in patients with HF [12–20]. They showed a significant correlation between RVEF and the parameters of CPET (peak VO_2_ and/or VE/VCO_2_ slope) and suggested the potential utility of CPET variables in risk stratification of patients with either moderate or advanced HF [15, 17, 19]. On the other hand, several studies did not find any significant correlations between the investigated parameters [13, 14, 16, 18]. Moreover, these findings were supported by the results of Witte et al. [19] and Rubis et al. [20] who also did not establish any significant correlations between tissue Doppler derived measures of long axis LV and RV function and exercise capacity in HF patients. These data support the fact that RNV and conventional echocardiography techniques of RV assessment were not able to demonstrate the correlation between RV function and exercise capacity, and stratify patients in relation to the severity of HF.

The recent introduction of two-dimensional speckle tracking echocardiography (2D- STE) and three–dimensional echocardiography (3DE) in clinical practice has implied great progress in HF management [21–23]. Speckle tracking imaging is a relatively novel method for the assessment of myocard deformation, and has many advantages (e.g. angle-independent, rapid, less load-dependent, more accurate) over the conventional echocardiography methods used for the evaluation of cardiac function and mechanics [21–23]. To date, only two studies [24, 25] have investigated the association between RV function assessed by 2D-STE and 3DE and exercise capacity in patients with ischemic dilated cardiomyopathy. Since the correlation between RV function and exercise capacity remains unclear, and needs to be further elucidated, this study aimed to investigate the correlation between 2D-STE and 3DE parameters of RV and exercise capacity in heart failure patients with reduced EF.

## Material and methods

### Study population

This cross-sectional study was conducted at the Department of Cardiology, University Hospital “Dr Dragisa Misovic” Belgrade, Serbia from February 2014 to February 2016. The study protocol was approved by the Ethics Committee of University Clinical Hospital Center "Dr. Dragiša Mišović - Dedinje" and Faculty of Medicine, University of Belgrade. Written informed consent was obtained from all participants after all procedures had been fully explained to them and prior to clinical and laboratory examinations. All procedures were conducted in accordance with the guidelines of the World Medical Association Declaration of Helsinki for human subjects.

The present study included 54 consecutive patients with diagnosed ischemic LV systolic dysfunction (HF with reduced EF <40%) and sinus rhythm [26] referred to our clinic for condition evaluation. Patients aged over 75, and those with severe angina syndrome, atrial fibrillation, severe valvular disease, anemia or chronic obstructive pulmonary disease were excluded from the study.

All patients underwent a physical examination, including anthropometric measures (height, weight), laboratory testing (creatinine, urea, uric acid (SUA), level of serum iron, hemoglobin, the fasting glucose level, glycated hemoglobin (HbA1c), total cholesterol, high and low density lipoprotein (HDL and LDL), triglycerides, C-reactive protein (CRP), N-terminal pro brain natriuretic peptide (NT-proBNP)), echocardiography, and CPET. Additionally, body mass index (BMI) was calculated for each patient. Based on the proposed values of analyzed CPET variables (VO_2,_ VE/VCO_2_) [27, 28] all participants were further subdivided into 4 subgroups: VO_2_ peak ≤ 15 ml/kg/min, VO_2_ peak > 15 ml/kg/min, VE/VCO_2_ slope < 36 and VE/VCO_2_ slope ≥ 36.

### Echocardiography

Echocardiographic examinations were performed using a 2.5MHz transducer with harmonic capability, whereas RT3DE data set acquisition of the right ventricle was obtained using a 3DE volumetric transducer of a Vivid 7 ultrasound machine (GE Healthcare, Horten, Norway).

#### Standard 2DE echocardiographic examination

The values of all 2DE echocardiographic parameters were calculated according to the current recommendations, i.e. as the average value of three consecutive cardiac cycles [29]. Left ventricular ejection fraction (EF) was estimated by using the biplane method. Pulsed Doppler measurements included the transmitral early diastolic peak flow velocity (E), and the ratio between mitral flow E peak velocity and tissue Doppler derived e’ of average septal and lateral mitral annulus (MV E/e’av).

#### Right ventricle and atrium

The right ventricular (RV) diameters were measured in the four-chamber apical view (basal, mid-cavity and longitudinal RV diameter) [30]. The RV fractional area change (FAC) was measured from the apical four-chamber view according to the guidelines [30]. Two-dimensional RV ejection fraction was calculated by modified Simpson’s rule [30]. The right atrial (RA) diameters were measured in the apical four-chamber view at the ventricular end-systole. Additionally, RA areas (RAA) and volumes (RAV) were obtained [30].

Tricuspid inflow (E) and tissue Doppler velocities (e’, s’) were evaluated in the 4-chamber view and E/e’ ratio was calculated. Tissue Doppler imaging was used to obtain the RV myocardial velocities in the apical four-chamber view with a sample volume placed at the lateral segment of the tricuspid annulus [30].

RV global systolic function was assessed as the tricuspid annular plane systolic excursion (TAPSE) [30]. Systolic pulmonary artery pressure (SPAP) was measured in a subset of patients with minimal/mild tricuspid regurgitation.

#### Two-dimensional strain

Two-dimensional strain imaging was performed by using three consecutive cardiac cycles in the apical four-chamber view [30]. The frame rate ranged between 60 and 80 frames/second. A commercially available software EchoPAC 110.1.2 (GE-Healthcare, Horten, Norway) was used for 2DE strain analysis. Thus, we determined global longitudinal strain of the right ventricle (RV GLS). RV GLS is assessed in the apical 4-chamber viewwith the myocardium of the entire RV being divided into six segments, so that each wall of the RV is divided into basal, middle, and apical segments. [7].

#### 3DE acquisition

A full-volume acquisition of the RV required for further analyses was obtained by harmonic imaging from an apical approach. Six electrocardiogram-gated consecutive beats were acquired during an end-expiratory breath-hold to generate a full volume. All data sets were stored digitally and analyzed by RV Tom Tec software (EchoPAC 110,1,2., GE-Healthcare, Horten, Norway). The frame rates ranged between 20 and 30 frames/second. The software was also used to measure RV end-diastolic volume (EDV), end-systolic volume (ESV), stroke volume (SV), and EF.

#### Cardiopulmonary exercise testing (CPET)

All patients underwent a maximum symptom-limited treadmill exercise test according to a modified Naughton protocol [31]. Each of the 6 levels lasted for 3 minutes with a constant speed of 3 km/h, and 0 elevation that was increased by 3, 5% after each interval. Patients were encouraged to continue with the test as long as their respiratory exchange ratio ≥ 1 [32]. The peak oxygen uptake (VO_2_), carbon dioxide production (VCO_2_), and minute ventilation (VE) were assessed by means of breath-by-breath gas analysis (CARDIOVIT CS-200 Ergo-Spiro system; Schiller AG, Baar, Switzerland). Spirometry was performed in all participants before the cardiopulmonary exercise testing, while forced expiratory volume was obtained in the first second. Forced vital capacity was also taken and calculated as a percentage of the predicted values for the patients’ age and gender. VO_2_ was defined as an average value within the last 20 seconds of exercise and expressed as ml/kg/min and METs (1 MET equals 3.5 ml of oxygen uptake per kilogram of body weight per minute). The ventilatory anaerobic threshold and oxygen uptake at this level was identified in all participants and expressed as a percentage of VO._2_. VE/VCO_2_ slope, which showed a linear increase of ventilation relative to carbon dioxide production, was automatically calculated using Schiller software.

### Statistical analysis

Statistical analysis was performed via IBM SPSS Statistics for Windows Software (Version 20.0, IBM Corp, Armonk, NY, USA) and R: A Language and Environment for Statistical Computing (Version 3.0.3, R Foundation for Statistical Computing, Vienna, Austria). The results were presented as counts (percentages), mean ± standard deviation, or median (minimum-maximum). Group comparisons were performed using Student’s t-test or Mann-Whitney U-test. The correlation between the two numerical variables was tested using Pearson and Spearman correlation analysis. The χ2 analysis was conducted to assess statistical significance between categorical data. Receiver operating characteristic (ROC) analysis was performed to determine the best echocardiographic parameter of RV systolic function in different subgroups. P-values lower than 0.05 were considered statistically significant.

## Results

The demographic and clinical parameters of the study population are presented in Table 1. There were no significant differences in the age and gender distribution between the examined subgroups (Table 1).

**Table 1:** Demographic characteristics and clinical parameters of the study population.

Variables	VO_2_ ml/kg/min		P value	VE/VCO_2_ slope		P value
	≤ 15 n = 17	>15 n = 37		<36 n = 37	≥36 n = 17	
Age (years)	64.3±7.9	62.6±8.1	0.487 ^a^	63.4±8.5	62.7±6.8	0.777 ^a^
Gender (M/F)	14/3	32/5	0.487 ^b^	32/5	14/3	0.696 ^b^
BMI (kg/m^2^)	26.8±3.7	26.3±3	0.582 ^a^	26.4±3.	26.5±3.6	0.951 ^a^
NT-pro BNP (pg/ml)	865 (19–5344)	403 (20–17582)	0.171^c^	308 (19–17582)	1705 (64–10367)	0.001 ^c^
Creatinine (umol/L)	85 (58–249)	87 (43–306)	0.737 ^c^	83 (43–260)	90 (58–306)	0.113 ^c^
Urea (mmol/L)	8.4 (4.3–19.9)	7.6 (3.3–66)	0.801 ^c^	7 (3.8–66)	8.9 (3.3–19.9)	0.157^c^
Uric acid (umol/L)	0.4±0.1	0.4±0.1	0.374 ^a^	0.4±0.1	0.5±0.1	0.015 ^a^
Fasting plasma glucose (mmol/L)	7.8±4.1	6.5±2.6	0.254 ^a^	6.3±2.4	8.3±4	0.065 ^a^
HbA1c (%)	6.8±1.5	6.3±1.3	0.250 ^a^	6.1±0.9	7.3±1.9	0.034 ^a^
Cholesterol (mmol/L)	4.8±1.2	4.9±1.2	0.908^a^	5.±1.2	4.5±1.1	0.142 ^a^
LDL (mmol/L)	3±1	2.9±0.9	0.729 ^c^	3±1	2.8±0.8	0.494 ^c^
HDL (mmol/L)	1.1±0.3	1.2±0.5	0.635 ^a^	1.2±0.5	1.1±0.3	0.273 ^a^
Triglycerides (mmol/L)	1.6 (0.6–4.2)	1.7 (0.2–6.2)	0.755 ^c^	1.4 (0.2–6.5)	1.1 (0.6–4.5)	0.188 ^c^
Level of serum iron (umol/L)	11.2±5.3	14.5±5.8	0.060 ^a^	14.9±5.	10.5±5	0.009 ^a^
Hemoglobin (g/L)	131.1±16.7	137.6±16	0.188 ^a^	139.5±14.5	126.8±17.2	0.007 ^a^
CRP (mg/L)	4 (1–117)	2.1 (1–34)	0.013 ^c^	2(1–32)	6. (1–117)	0.001 ^c^

Data are presented as mean ± standard deviation, median (minimum and maximum) or n-number of participants. M–male, F–female, BMI–body mass index, NT-pro BNP–N-terminal pro brain-type natriuretic peptide; LDL–low-density lipoprotein, HDL–high-density lipoprotein; CRP–C reactive protein; HbA1c –glycated hemoglobin

^a^ Student’s t-test

^b^ Fisher’s Exact test

^c^ Mann-Whitney U-test.

The median CRP level was significantly higher in the subgroup of patients with peak VO_2_ ≤ 15 ml O_2_/kg per min compared to the subgroup of patients with peak VO_2_ > 15 ml O_2_/kg per min (*p* = 0.013) (Table 1). Furthermore, the levels of CRP, NT-pro BNP, uric acid, and HbA1c were significantly higher in the subgroup of subjects with a VE/VCO_2_ slope ≥ 36 compared to the subgroup of subjects with a VE/VCO_2_ slope <36 (*p* = 0.001, *p* = 0.001, *p* = 0.015, *p* = 0.034, respectively). On the other hand, the levels of serum iron and hemoglobin were significantly lower in the subgroup of subjects with a VE/VCO_2_ slope ≥36 compared to the subgroup of subjects with a VE/VCO_2_ slope <36 (*p* = 0.009, *p* = 0.007, respectively) (Table 1).

The conventional 2DE parameters in the investigated subgroups are listed in Table 2. RVFAC was significantly decreased, while RVEF and MV E/e’ were significantly increased in the subgroups of patients with peak VO_2_ ≤15 ml O_2_/kg per min compared to the subgroups of subjects with peak VO_2_≥15 ml O_2_/kg per min (*p* = 0.036, *p* = 0.005, *p* = 0.013, respectively) (Table 2). Furthermore, RV basal diameter, SPAP, TAPSE, TV E/e’, EF biplane, and MV E/e’ were significantly increased in the subgroups of patients with a VE/VCO_2_ slope ≥36 compared to the subgroups of patients with a VE/VCO_2_ slope ≤36 (*p* = 0.024, *p* = 0.002, *p* = 0.017, *p* = 0.023, *p* = 13*p* = 0.001, respectively) (Table 2).

**Table 2:** Conventional two-dimensional echocardiography parameters in the investigated groups.

Variables	VO_2_ ml/kg/min		*P* value *	VE/VCO_2_ slope		*P* value*
	≤ 15 n = 17	>15 n = 37		<36 n = 37	≥36 n = 17	
**Right ventricle**						
Basal diameter (mm)	37.4±6.2	36.6±5.1	0.618	35.8±5.1	39.3±5.6	0.024
Mid–RV diameter (mm)	28.3±6.2	27.2±4.9	0.489	26.6±5.2	29.5±5.2	0.064
Longitudinal diameter (mm)	62±6.9	59.5±8	0.261	59.8±8.3	61.4±6.3	0.468
Diastolic volume (mL)	87.9±23.8	93.8±15.7	0.283	91±19	94.1±18.1	0.573
Systolic volume (mL)	43±10.6	43.7±8.4	0.792	42.4±8.7	45.8±9.7	0.211
RV fractional area change (%)	36.3±5.7	40.1±7.1	0.036	30.2±6.5	37.1±7.4	0.124
RV ejection fraction (%)	51.1±7.6	53.2±5.8	0.270	53.2±6.1	51.2±7.2	0.314
SPAP (mmHg)	42.5±16.2	29.2±9.2	0.005	28.9±10	43±14.6	0.002
TAPSE (mm)	1.1±1	1.1±0.6	0.522	1.1±0.07	1.1±0.08	0.017
TV E/e’	0.9±0.1	0.9±0.1	0.252	0.8±0.1	0.9±0.1	0.023
**Right atrium**						
Transverse diameter (mm)	37.8±7.6	35.6±5	0.210	35.3±4.7	38.4±8	0.149
Longitudinal diameter (mm)	51.9±6.1	50.9±5.6	0.553	50.5±5.5	52.8±5.9	0.161
Area (cm^2^)	15.8±4.2	15.2±2.6	0.543	15±2.6	16.2±4	0.205
Volume (mL)	40.7±20	37.8±9.8	0.476	36.5±9.8	43.6±18.7	0.070
**Left ventricle**						
EF biplane (%)	31.6±6.5	35±6.1	0.052	35.5±6	30.1±6.4	0.013
MV E/e’	1.1±0.2	1±0.2	0.013	0.9±0.2	1.1±0.2	0.001

The data are presented as mean ± standard deviation, RV–right ventricle, SPAP–systolic pulmonary artery pressure, TAPSE–tricuspid annular plane systolic excursion, TV E/e’—ratio between tricuspid inflow (E) and tissue Doppler derived e’ of the lateral tricuspid annulus, MV E/e’–ratio between mitral flow E peak velocity and tissue Doppler derived e’ of the septal mitral annulus, EF–ejection fraction.

* Student’s t-test.

The analysis of 2D STE and 3DE parameters revealed that 2D RVGLS was significantly lower in the subgroups of patients with peak VO_2_ ≤15 ml O_2_/kg per min compared to the subgroups of subjects with peak VO_2_ ≥15 ml O_2_/kg per min (*p* = 0.028 (Table 3). Additionally, 2D RVGLS, 3D RV SV and 3D RV EF were significantly lower, while 3D RV ESV was significantly higher in the subgroups of patients with a VE/VCO_2_ slope ≥36 compared to the subgroups of patients with a VE/VCO_2_ slope <36 (*p* = 0.001, *p* = 0.001,*p* = 0.004, *p* = 0.001, respectively) (Table 3).

**Table 3:** Two–and three-dimensional speckle tracking echocardiography parameters in the investigated groups.

Variables	VO_2_ ml/kg/min		*P* value *	VE/VCO_2_ slope		*P* value*
	≤ 15 n = 17	>15 n = 37		<36 n = 37	≥36 n = 17	
2D RVGLS (%)	19.4±3.1	21.3±2.9	0.028	21.6±2.7	18.6±2	0.001
3D RV EDV (mL)	114.5±13.7	109.2±15.6	0.230	111.2±15.7	110.1±14.1	0.798
3D RV ESV (mL)	62.2±8.6	54.7±11.1	0.017	53.8±10	64.1±9.6	0.001
3D RV SV (mL)	52.2±13.8	54.3±13.6	0.612	57.1±12.3	45.9±13.1	0.004
3D RV EF (%)	45.2±8	49.6±8.7	0.083	51.4±6.9	41.4±8.2	0.001

Data are presented as mean ± standard deviation, 2D –two dimensional, RVGLS–global longitudinal strain of right ventricle, 3D –three-dimensional, RV EDV–right ventricle end-diastolic volume, ESV–end-systolic volume, SV–stroke volume, EF–ejection fraction.

* Student’s t-test

**Fig 1:**
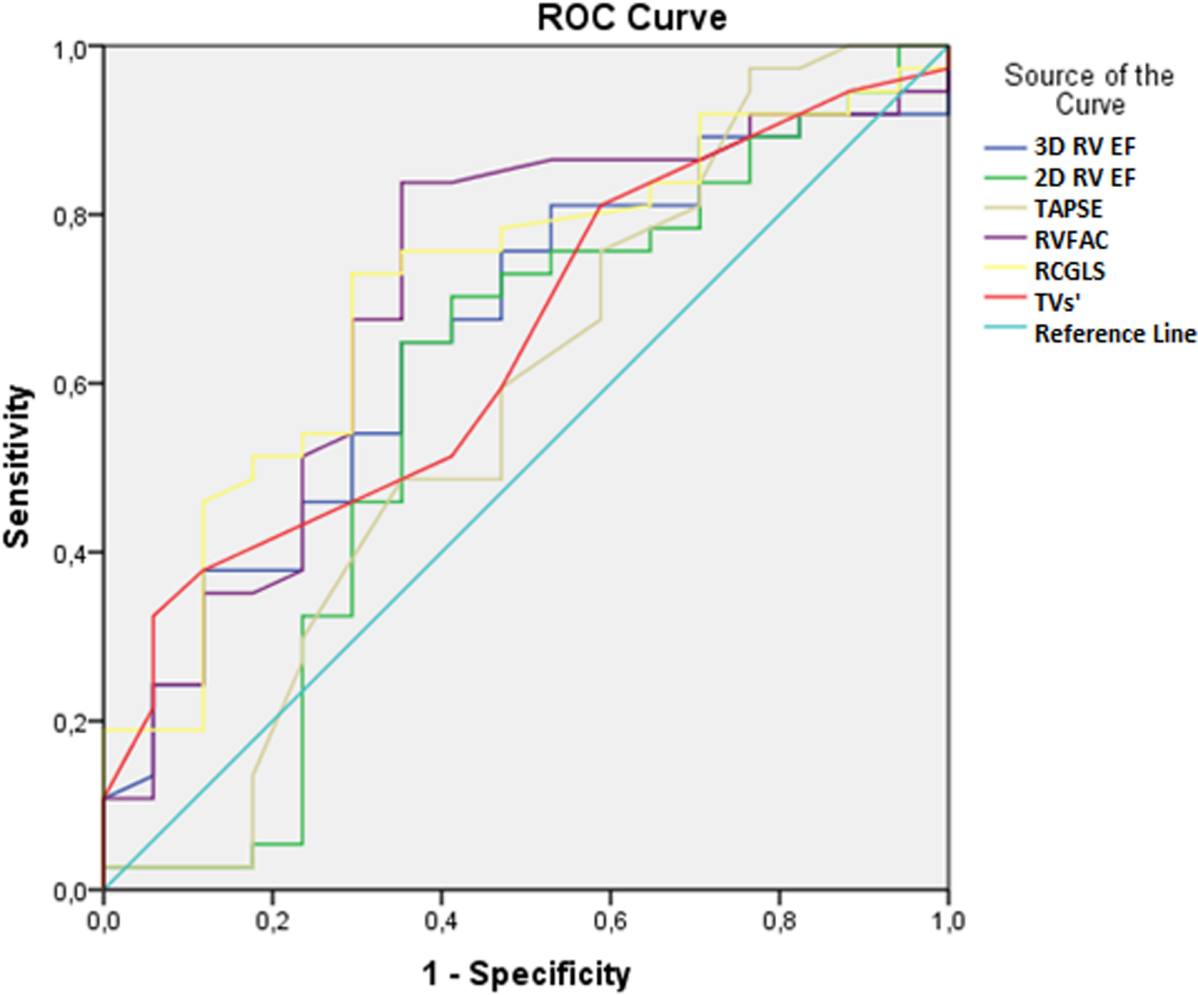
Receiving operating characteristics (ROC) curve for the selected echocardiographic parameters in relation to the peak VO2 values.

**Fig 2:**
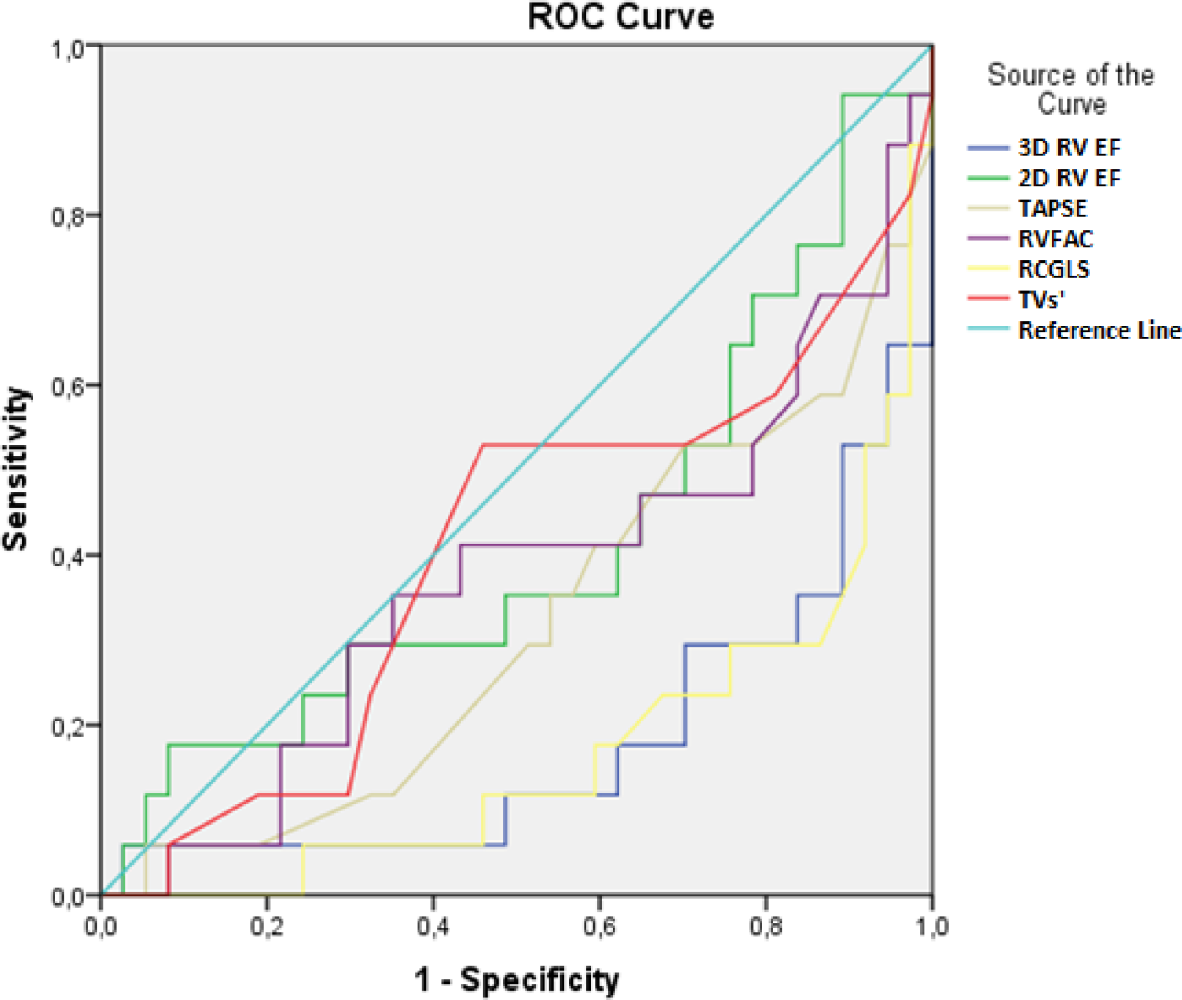
Receiving operating characteristics (ROC) curve for the selected echocardiographic parameters in relation to the VE/VCO2 slope values.

The impact of peak VO_2_ and VE/VCO_2_ slope values on the echocardiographic parameters was further investigated by ROC analysis. The ROC curve areas for the selected echocardiographic parameters in relation to the peak VO_2_ values are presented in Fig 1 and Table 4. The highest area under the ROC curve was observed for RVGLS 0.71 [95% confidence interval (CI) 0.56–0.86] (*p* = 0.014). Moreover, the ROC curve areas for the selected echocardiographic parameters in relation to the VE/VCO_2_ slope values are presented in Fig 2 and Table 5. The highest area under the ROC curve was also observed for RVGLS 0.83 [95% CI 0.71–0.95] (*p* = 0.001).

**Table 4:** Receiving operating characteristics (ROC) curve for the selected echocardiographic parameters in relation to the peak VO2 values.

Variables	Area	SE	95% CI		P value
			Lower bound	Upper bound	
3D RV EF (%)	0.657	0.078	0.504	0.811	0.065
2D RV EF (%)	0.582	0.093	0.400	0.763	0.337
TAPSE (%)	0.572	0.092	0.392	0.752	0.397
RVFAC (%)	0.705	0.079	0.551	0.860	0.016
RVGLS (%)	0.709	0.075	0.562	0.856	0.014
TVs’	0.648	0.078	0.495	0.801	0.083

SE–standard error, CI–confidence interval, 3D RV EF–three-dimensional right ventricle ejection fraction, 2D RV EF–two-dimensional right ventricle ejection fraction, TAPSE–tricuspid annular plane systolic excursion, RVFAC–right ventricle fractional area change RV GLS–right ventricle global longitudinal strain, TVs’–tissue Doppler derived tricuspid lateral annular systolic velocity.

**Table 5:** Receiving operating characteristics (ROC) curve for the selected echocardiographic parameters in relation to the VE/VCO2 slope values.

Variables	Area	SE	95% CI		P value
			Lower bound	Upper bound	
3D RV EF (%)	0.820	0.067	0.688	0.952	0.001
2D RV EF (%)	0.587	0.088	0.413	0.760	0.310
TAPSE (%)	0.682	0.080	0.525	0.839	0.033
RVFAC (%)	0.626	0.087	0.455	0.7596	0.141
RVGLS (%)	0.831	0.061	0.712	0.951	0.001
TVs’	0.594	0.088	0.421	0.766	0.272

SE–standard error, CI–confidence interval, 3D RV EF–three-dimensional right ventricle ejection fraction, 2D RV EF–two-dimensional right ventricle ejection fraction, TAPSE–tricuspid annular plane systolic excursion, RVFAC–right ventricle fractional area change RV GLS–right ventricle global longitudinal strain, TVs’–tissue Doppler derived tricuspid lateral annular systolic velocity.

Significant positive and negative correlations were observed between the investigated CPET and echocardiographic parameters (Table 6).

**Table 6:** Correlation between the parameters of cardiopulmonary exercise testing and echocardiographic parameters.

Variables	VO_2_ ml/kg/min	VE/VCO_2_ slope
RV basal diameter (mm)	-.09	.259
Mid–RV diameter (mm)	.04	.130
RV longitudinal diameter (mm)	-.109	.204
RV diastolic volume (mL)	.192	.134
RV systolic volume (mL)	.122	.132
RV fractional area change (%)	.274 *	-.129
RV ejection fraction (%)	.134	-.017
SPAP (mmHg)	-.483 **	.466 **
TAPSE (mm)	.293 *	-.341 *
RA transverse diameter (mm)	.286 *	.245
RA longitudinal diameter (mm)	- .184	.124
RA area (cm^2^)	- .063	.093
RA volume (mL)	-.125	.168
EF biplane (%)	.347 *	-.452 **
MV E/e’	.275*	-.355 *
2D RVGLS (%)	.332 *	-.394 **
3D RV EDV (mL)	.008	.025
3D RV ESV (mL)	-.279 *	.417 **
3D RV SV (mL)	.2^46^	-.314 *
3D RV EF (%)	.335 **	-.450 **

* p < 0.05

** p < 0.001, RV–right ventricle, SPAP–systolic pulmonary artery pressure, TAPSE–tricuspid annular plane systolic excursion, RA–right atrium, EF–ejection fraction, MV E/e’–ratio between mitral flow E peak velocity and tissue Doppler derived e’ of the septal mitral annulus, 2D –two dimensional, GLS–global longitudinal strain, 3D –three dimensional, EDV–end-diastolic volume, ESV–end-systolic volume, SV–stroke volume.

## Discussion

Previous studies have confirmed that RV systolic dysfunction is a powerful prognostic variable in patients with HF [4–6]. Traditionally, it has been measured by obtaining nonvolumetric conventional echocardiographic parameters such as TAPSE or RVFAC [33, 34]. It has also been reported that they could be used both for the initial diagnosis and for the follow-up of RV function [6, 35]. Moreover, TAPSE and RVFAC have been shown to correlate with RVEF as measured by CMR or RNV [36]. In this study, the values of RVFAC were significantly decreased (p = 0.036), whereas the values of TAPSE were significantly increased (p = 0.017) in HF patients with a poor prognosis (peak VO_2_ ≤ 15 ml O_2_/kg per min and VE/VCO_2_ slope ≥ 36). Yet, RVFAC and TAPSE positively correlated with peak VO_2_, and there was also a significant negative correlation between TAPSE and VE/VCO_2_ slope. Although they are easy to obtain, reproducible, and have been validated in patients with HF, they only assess a regional RV function [36, 37]. Furthermore, TAPSE is relatively load–and angle–dependent, and is subject to cardiac translation. Of note, the main limitation in obtaining fractional linear shortening is the poor definition of the RV anterior wall [7].

RVEF assessed by conventional echocardiography has long been recognized as an important predictor of the outcome in HF patients [15]. In this study, the assessment of RVEF, RV diastolic and systolic volumes showed no significant difference between the investigated subgroups and there was no significant correlation with the investigated CPET parameters. The employment of conventional echocardiography in the assessment of RV volumes and RVEF is challenging, owing to the position of the RV in the chest and its complex geometry [21–23]. However, the introduction of new echocardiographic methods has significantly improved our ability to assess RV volumes and function. It is well known that 2D-STE and 3DE are angle-independent, rapid, less load-dependent, and more accurate compared to conventional echocardiography [21–23]. They enable accurate evaluation of RV volumes and RVEF with results that are similar to CMR measurements [38, 39]. Although CMR allows highly reproducible 3D quantification of RV and is regarded as the gold standard for the estimating RV function, there are several limitations related to its everyday clinical practice. In other words, it is not widely available, it can be time-consuming to perform, it is costly, and it is contraindicated in some subjects [39]. Given these facts, new echocardiographic methods represent the first choice for the evaluation of the RV structure, function and mechanics in HF patients [21–23].

This study employed 2D-STE and 3DE to determine whether new echocardiographic parameters of RV correlate with the exercise capacity of patients with reduced HF, and whether they are able to stratify these patients according to the values of peak VO_2_ (≤15, >15) and the VE/VCO_2_ slope (<36, ≥36). We showed that 2D RV GLS was significantly decreased in both subgroups of patients with a poor HF prognosis (p = 0.028, p = 0.014, respectively). The significance of these findings is supported by the fact that, in relation to the two investigated CPET parameters, the highest area under the ROC curve was observed for 2D RV GLS (p = 0.014, and p = 0.001). Yet, we observed a significant positive correlation between the peak VO_2_ values and 2D RV GLS, 3D RV SV, and 3D RV EF, while there was a significantly negative correlation between the VE/VCO_2_ slope values and the same group of parameters.

Our results are in accordance with the results of previously published studies [40–42]. Namely, Cameli et al. [40] showed that 2D RV GLS was the strongest predictor of the clinical outcome in 98 consecutive patients with advanced HF referred for cardiac transplant evaluation. Garcia-Martin et al. [41] reported that 2D RV GLS was a stronger predictor of HF development than TAPSE. Moreover, Vizzardi et al. [42] revealed that 2D RV GLS was the only variable associated with cardiac death or HF hospitalization, and it was a stronger predictor of an adverse outcome than TAPSE or RVFAC. It is important to emphasize that our results are also in agreement with the studies of D’Andrea et al. [25] and Salermo et al. [24]. Salermo et al. [24] evaluated the relationship between RV dysfunction and the response to CPET in patients with ischemic dilated cardiomyopathy. They detected a significant correlation among 2D RV GLS and VO_2_ peak (p<0.001) and stated that 2D RV GLS had remained significant even in multivariate analysis. D’Andrea et al. [25] reported that 3D RV EF directly correlated with VO_2_ peak (p<0.0001) and inversely with VE/VCO_2_ slope (p<0.001). Additionally, they reported that by multivariate analysis 3D RV EF had emerged as the independent determinant of VO_2_ peak % during CPET [25].

It is well known that the RV plays a pivotal role in the regulation and compliance of the pulmonary vascular system. With exercise, RV dysfunction results in the increase of vascular pressures and impairment of oxygen exchange. Based on these grounds, the superior prognostic strength of RV GLS with respect to other echocardiographic parameters could be explained by the fact that reduced RV strain more accurately reflects severe impairment of the RV performance. In fact, the systolic shortening of the RV from base to apex provides information on both RV emptying and dilation.

Analysis of clinical parameters revealed that the levels of CRP were significantly increased in HF patients with a poor prognosis. These results are in line with the previously reported findings [43] and suggest that these patients are associated with an increase in inflammation, which could be a consequence of ischemic necrosis that may have initiated this potent inflammatory stimulus. Furthermore, we found significantly higher values of creatinine, urea, and uric acid in HF patients with VE/VCO_2_ slope ≥36 compared to HF patients with VE/VCO_2_ slope < 36. These results imply a deterioration of renal function in patients with HF, which is also in agreement with previous reports [44–46]. Renal dysfunction (RD) is a frequent comorbid condition and a major determinant of the outcomes in patients with HF [44–46]. It has been reported that HF and RD may worsen each other through multiple mechanisms such as fluid overload and increased venous pressure, hypo-perfusion, neurohormonal and inflammatory activation, and concomitant treatment [44–46]. In our study, the levels of NT-pro BNP were significantly higher in HF patients with a poor prognosis. NT-pro BNP is secreted from the heart in response to cardiac hemodynamic stress mediated by volume and/or pressure overload [47, 48]. A wealth of previous studies have also reported that plasma concentrations of NT-pro BNP are increased in patients with HF [47, 48], which is in line with our findings. Although our results provide further evidence that the RV function can be noninvasively and objectively measured by 2D-STE and 3DE in heart failure patients with reduced ejection fraction our study has several limitations. The first one is related to small sample size. Based on inclusion criteria we finally included 54 heart failure patients with reduced EF (<40%). Moreover, it should be recognized that basal echo parameters do not completely reflect exercise and functional capacity of included patients. Therefore, further longitudinal studies with larger study groups and stress-echo analysis are required to confirm our results.

## Conclusion

In conclusion, the results of this study confirm previous observations and provide further evidence that the RV function can be noninvasively and objectively measured by 2D-STE and 3DE. The observed significant correlation between the examined parameters suggests that 2D RV GLS and 3D RV EF, SV are associated with exercise capacity in patients with reduced HF.
